# Advancements of 2D Materials-Based Membranes

**DOI:** 10.3390/membranes12010052

**Published:** 2021-12-31

**Authors:** Zakarya Othman, Khaled A. Mahmoud

**Affiliations:** Qatar Environment and Energy Research Institute (QEERI), Hamad Bin Khalifa University (HBKU), Doha P.O. Box 34110, Qatar; zothman@hbku.edu.qa

Our environment desperately needs creative solutions to limit the effect of industrialization’s fast rise and, consequently, to remediate vast amounts of harmful by-products and toxic exhausts. Day by day, nanomaterials are becoming a more viable and eco-friendly option for efficient environmental remediation. Due to their large surface area, high reactivity, enhanced redox, and photocatalytic properties, a variety of nanomaterials are identified as ideal adsorbents, catalytic and antibacterial agents, as well as functional membranes. Among the emerging nanomaterials, 2D nanomaterials have attracted increasing attention recently following the discovery of graphene, which quickly placed among the most promising materials due to its distinctive structures and novel properties. Moreover, the discovery of graphene has paved the way for the synthesis of various 2D materials. Around 20 different types of 2D nanomaterials have been reported to date, including graphene, MXenes, graphitic carbon nitride (g-C_3_N_4_), black phosphorus (BP), metal phosphorus trichalcogenides, hexagonal boron nitride (h-BN), transition metal dichalcogenides (TMDs), and 2D polymers. As a result of the quantum confinement effects, and the dependence on material’s composition, atomic arrangement, and layer thickness, these 2D nanomaterials display an array of exceptional electronic, optical, physical, and chemical properties that are quite unique. Therefore, by developing advanced, functional 2D nanomaterial-based membranes, we may achieve a new level of efficiency, selectivity, sensitivity, and mechanical stability, all of which are critical for environmental remediation applications.

This Special Issue discusses critical themes in the design and manufacturing of advanced 2D nanomaterial-based membranes, their functionalization, and their applications in specialized separation processes such as wastewater treatment, and desalination. This Special Issue accumulated a total of six articles, including four research papers and two reviews. This collection of papers and reviews focuses on the synthesis, design, optimization, and applications of advanced 2D nanomaterial-based membranes, as well as on current developments and future prospects in this field.

The ability of nano-engineered MXene–graphene composites to provide improved mechanical characteristics (e.g., tensile strength and elasticity), deep electrical conductivity, and very rich surface chemistries (e.g., the coexistence of hydrophilic and hydrophobic surfaces) have piqued researchers’ attention for water purification applications. Berdiyorov et al. [1] have used first-principles density functional theory calculations to investigate the structural properties and strength of interlayer interactions of various heterostructure compositions of MXene with graphene and silver nanoparticles for the purpose of improving the structural properties of MXene membranes for water purification applications. Owing to the profound variations of electrostatic potential across the heterostructure layers, the MXene–graphene hybrid bilayer configuration revealed stronger interlayer interactions as compared to graphene/graphene and MXene–MXene bilayers. Density functional theory calculations also showed that decorating MXene with Ag nanoparticles increased the binding energy of stacked MXene layers, which may be beneficial for improving the operational stability of MXene membranes used in water treatment applications.

Desalination via pervaporation is one of the membrane-based separation techniques that require membranes with exceptional chemical and thermal stability. In the paper by Almarzooqi et al. [2], the stability and separation performance of novel graphene oxide–polyethersulfone membranes, prepared by vacuum filtration of a graphene oxide suspension on polyethersulfone and cross-linked using Zn^2+^, have been evaluated against brackish water and produced water. Following 72 h of pervaporation, the examined membranes revealed a drop in Zn^2+^ content, indicating partial hydrolysis of the Zn^2+^ crosslinker that held the GO sheets together. While salt was rejected in all instances, soluble organic components (phenol, cresol, and naphthenic acid) were rejected at a rate of 40–50%.

Additionally, 2D nanomaterials have also been employed in the fabrication of 3D porous structures. N’Diaye et al. [3] have employed a porous foam composite (GO–PEI–FeNPs), synthesized by reducing graphene oxide layers and co-precipitating iron in a hydrothermal system using polyethyleneimine as a crosslinker, in removing high molecular weight contaminants such as bisphenol A, progesterone, and norethisterone. Findings revealed a significant adsorption capacity of GO–PEI–FeNPs foam, with maximum adsorption percentages of 68%, 49%, and 80% for bisphenol A, progesterone, and norethisterone, respectively. Additionally, the inclusion of magnetic iron nanoparticles has facilitated the separation process after decontamination. 

In the work of Chergui et al. [4], an rGO–PEI–Flu-DEX-apt graphitic foam membrane, composed of reduced graphene oxide, polyethyleneimine, and dexamethasone-aptamer, was employed as a selective membrane for the removal of dexamethasone hormone from water. The fluorescence quenching properties of graphene oxide were employed to track and validate the membrane adsorption of the analyte. Different graphene oxide concentrations have been used in the preparation of these foam membranes. Findings revealed that higher selectivity could be achieved by increasing the amount of GO, which has been shown to affect both the distribution of foam pores and the amount of DNA that can be loaded into those pores. Additionally, in the presence of other steroid hormone analogs such as progesterone, estrone, estradiol, and 19-norethindrone, the rGO–PEI–Flu-DEX-apt membrane demonstrated high sensitivity and specificity toward the dexamethasone hormone.

Due to their unique structural features and precise molecular sieving ability, 2D nanomaterials are intriguing candidates for effective organic solvent nanofiltration (OSN) membranes. In the review by Nam et al. [5], the focus was to describe the most current methods for fabricating nanoporous graphene (NG)-based membranes, including single-layer NG, multilayer graphene laminates, and multilayer NG laminates, and their applications in OSN processes. Additionally, techniques for generating nanopores on graphene were explored, as well as the problems related to pore engineering and opportunities associated with the commercialization of NG membranes.

Using advanced 2D nanomaterials (2DNMs) in membranes manufacturing is comprehensively summarized in the review of Shahzad et al. [6]. Several 2DNM systems embedded within different membrane sructures are critically reviewed, especially graphene family materials, carbon nanotubes (CNTs), and MXenes. Several factors affecting membranes performance such as membrane fouling causes, tyepes, and anti-fouling techniques were discussed. With the help of this review, researchers may better understand how to design next-generation 2DNMs, which will allow to develop selective, multifunctional, programmable membranes for different environmental applications. Additionally, a perspective on the urgent constraints and future prospects in the synthesis and implementation of 2DNM-enabled membranes for wastewater treatment is presented.

In the research presented in these six papers, 2D nanomaterials have been shown to have an essential role in improving the performance of different membrane-based separation processes. The writers of these contributions have dived into the major features of 2D nanomaterials and addressed several constraints. Greater knowledge of the recent advancements of various 2D nanomaterial-based membranes and the potential for filling research gaps has been gained. This special issue is expected to provide some useful recommendations and future directions for creating more fascinating and high-performance 2D nanomaterial-based membranes.
